# Racial/ethnic and gender differences in smoking in early middle adulthood

**DOI:** 10.1016/j.ssmph.2022.101119

**Published:** 2022-05-09

**Authors:** Juhee Woo, Elizabeth Lawrence, Stefanie Mollborn

**Affiliations:** a Appalachian State University, Department of Sociology, ASU Box 32115, 209 Chapell Wilson Hall, 480 Howard Street, Boone, NC, 28608, USA; b University of Nevada, Las Vegas, Department of Sociology, CBC-B 243, Las Vegas, USA; c Stockholm University, Department of Sociology, SE-106 91, Stockholm, Sweden; d University of Colorado Boulder, Institute of Behavioral Science, UCB 483, Boulder, CO 80309-0483, USA

## Abstract

Research has documented important differences in smoking rates across race/ethnicity, gender, and age. Much of the research has either focused on smoking initiation among adolescents or cessation among adults, but little is known about racial/ethnic patterns in intermittent and daily smoking across young and early middle adulthood. We therefore use the life course perspective to identify how racial/ethnic and gender differences in smoking unfold across adulthood. Analyses investigate whether racial/ethnic and gender differences exist in the likelihood of daily smoking in early middle adulthood and whether these disparities persist after the inclusion of adolescent and early midlife sociodemographic characteristics and young adult smoking patterns. Descriptive statistics and multivariate binary logistic regression analyses employ recent data from a nationally representative sample of adults using the National Longitudinal Study of Adolescent to Adult Health (Add Health; N = 8,506). We find evidence that life course patterns of smoking differ across race/ethnicity and gender subgroups. In early middle adulthood (ages 33–44), White women are more likely to smoke daily than Black or Hispanic women. In contrast, there are no significant differences between White and Black men, but White men are more likely to smoke daily than Hispanic men. These racial/ethnic differences are no longer significant for men when previous smoking is controlled, suggesting that early young adult smoking plays an important role in the development of smoking disparities across race/ethnicity. Further, we find that young adult intermittent smoking is associated with daily smoking in early midlife, and this relationship is stronger for Black, compared to White, men and women. Although Black women display lower odds of daily smoking in early midlife compared to White women, they exhibit a higher risk of transitioning from intermittent to daily smoking. These results highlight the importance of considering a greater diversity of life course patterns in smoking across race/ethnicity and gender in future research and policies.

## Highlights

• Life course patterns of smoking differ across race/ethnicity and gender subgroups.
• Racial/ethnic differences in early midlife daily smoking persist beyond adolescent and concurrent sociodemographic factors.
• Early young adult smoking plays an important role in the development of smoking disparities across race/ethnicity.
• Smoking research and policies should consider a greater diversity of life course patterns in smoking.

## 1 Introduction

Cigarette smoking remains a leading preventable cause of death in the United States (US Department of Health and Human Services, 2014). Although the proportion of cigarette smokers has declined over time, approximately 14% of adults (34.2 million) smoke cigarettes (Creamer et al., 2019). Cigarette smoking has diffuse health consequences, resulting in hundreds of thousands of excess deaths annually (Lariscy et al., 2018). Additionally, smoking is a key contributor to health disparities. Its prevalence differs across many social characteristics, including race/ethnicity, gender, socioeconomic status (SES), and geographic location (Drope et al., 2018).

Studies focusing on developmental trajectories of smoking initiation and cessation show differences across individual and contextual characteristics. Initiation in adolescence (12–17) and early young adulthood (18–24) and cessation across adulthood demonstrate heterogeneity by race/ethnicity and gender. Non-Hispanic Whites and Hispanics generally start smoking in adolescence or early young adulthood, whereas non-Hispanic Blacks have lower adolescent smoking rates but increasing rates in young adulthood (e.g., Kandel et al., 2011; Lawrence et al., 2014; Pampel, 2008). How these patterns unfold in early middle adulthood across race/ethnicity and gender is not yet known.

The role of intermittent smoking is also an open question. Researchers have generally characterized intermittent smoking as “social,” linking the behavior to a broader young adult lifestyle that may include other risky behaviors and is not expected to continue as youths mature (See Schane et al., 2010 for a systematic review on light and intermittent smoking). However, given recent increases in intermittent smoking (Schane et al., 2010) and differences in smoking trajectories across race/ethnicity and gender, intermittent smoking may matter for racial/ethnic and gender smoking patterns.

Our study therefore documents the likelihood of daily smoking across race/ethnicity and gender in early middle adulthood, determining if and how the timing of prior intermittent and daily smoking shapes these patterns. We present smoking pathways across different racial/ethnic and gender groups entering early middle adulthood using longitudinal data from the National Longitudinal Study of Adolescent to Adult Health (Add Health). We analyze data from adolescence, early and late young adulthood, and the newly released Wave V, when respondents were transitioning to early middle adulthood (ages 33–44).

### 1.1 Theoretical perspectives

Our study is guided by two theoretical perspectives: the life course and intersectionality. Within their life course, people follow social timing, or “the incidence, duration, and sequence of roles” and “relevant social expectations and beliefs based on age” (Elder, 1994:6). Thus, historical time and contexts that guide individuals’ life courses, different social timings, social connectivity, and decisions influence their health behaviors and outcomes. A life course approach to smoking can illustrate how disparities emerge over age and how social factors influencing smoking are age-dependent (Maralani, 2013). Although the life course perspective discusses how earlier social conditions and statuses shape later health, it does not elaborate how intersecting social statuses create varied experiences and inequalities across time. We therefore incorporate an intersectional lens.

Intersectionality is a theoretical framework for understanding how multiple social identities, such as race, gender, and SES, intersect at the micro-level of individual experience to reﬂect interlocking systems of oppression (e.g., racism, sexism, heterosexism, classism) at the macro social-structural level (Crenshaw, 1991). We use an intersectional perspective to examine how racial/ethnic differences in smoking intersect with gender, and how age-patterned smoking trajectories differ by racial/ethnic and gender subgroup. Therefore, we integrate life course and intersectional approaches to understand how intersecting social identities, including gender, race/ethnicity, and life course stage, are associated with differences in smoking.

### 1.2 Racial/ethnic and gender patterns over age

Smoking prevalence varies across racial/ethnic groups. The Centers for Disease Control and Prevention (CDC; Creamer et al., 2019) reports that among U.S. adults, non-Hispanic American Indians/Alaska Natives (22.6%) and non-Hispanic multiracial individuals (19.1%) are the two racial/ethnic groups with the highest smoking rates. Non-Hispanic Whites (15%) and non-Hispanic Blacks (14.6%) have lower yet still substantial smoking rates, followed by Hispanics (9.8%) and non-Hispanic Asians (7.1%).

These overall averages, however, belie substantial life course differences. Previous studies have documented an “age crossover” pattern, or the age-related reversal with the prevalence of current smoking being lower among non-Hispanic Blacks than Whites in adolescence but higher in adulthood. Smoking initiation in early life is higher among Whites than Blacks (Escobedo et al., 1990), and the average age of smoking onset is earlier for Whites than Blacks (Harrell et al., 1998). Black youths also exhibit lower likelihoods of progression to daily smoking (Kandel et al., 2004), weekly smoking (Ellickson et al., 2003), or current smoking (Flint et al., 1998) from smoking onset. However, they lose their advantage relative to Whites as they approach and enter their thirties (Lawrence et al., 2014). Research attributes this crossover to later smoking initiation among African Americans (Kandel et al., 2011) and greater cessation among Whites in older ages (King et al., 2004; Pampel, 2008). Little is known about how these trajectories unfold in early middle adulthood.

Gender further complicates these patterns. Smoking prevalence and initiation age are somewhat similar for adolescent girls and boys, yet girls are less likely to smoke heavily but also progress faster from their first cigarette to daily smoking (Fulkerson & French, 2003; Thorner et al., 2007). The gender gap grows with age, and by young adulthood, smoking prevalence is substantially higher for men (see Lawrence et al., 2014; Pampel, 2008; Pampel et al., 2014).

Little research examines smoking in early middle adulthood across the intersection of race/ethnicity and gender. Yet, smoking may cause greater health risks in midlife due to new health conditions and risk factors emerging in midlife. Middle adulthood often brings greater life course stability and security in SES, work, and family, thus making age-by-age differences potentially less important when examining smoking patterns. At the same time, middle adulthood has become more dynamic (e.g., high divorce and re-partnering rates), and middle-aged adults are highly embedded in social relationships with aging parents, children, work colleagues and others. These relationships can buffer or exacerbate the daily stresses of midlife (Harris & McDade, 2018; Yang et al., 2016; Yang et al., 2014), which in turn may shape smoking patterns. Thus, we seek to document how smoking in early middle adulthood is patterned across intersections of race/ethnicity and gender.

### 1.3 Smoking patterns

We focus on daily smoking based on research demonstrating that it predicts greater health risks than less frequent smoking and following prior studies on racial/ethnic and gender differences in daily smoking (e.g., Hill et al., 2005; Hu et al., 2006; Kandel et al., 2004; Trinidad et al., 2009). Daily smokers are more likely to be nicotine-dependent (Van De Ven et al., 2010) and less likely to attempt to or successfully quit (Berg et al., 2012; Tindle & Shiffman, 2011). However, smoking patterns are complex, and researchers operationalize smoking categories in a number of ways. Defining and labeling “light or intermittent smokers” is especially challenging across ages, data sources, and research aims (Husten, 2009). However, researchers consistently consider smokers who smoke “nondaily,” “some days,” or “occasionally” as intermittent smokers, a pattern of smoking that is growing in prevalence and social significance (Husten, 2009; Schane et al., 2010; Trinidad et al., 2009).

Although intermittent smoking can represent chronic cigarette use (Hassmiller et al., 2003; Shiffman, 2009), it is often perceived as a transitional phase (between nonsmoking and daily smoking or vice versa; White et al., 2009; Zhu et al., 2003) or a temporary period of experimentation or smoking in social settings ending with cessation (Shiffman et al., 2015). There is not yet empirical consensus whether intermittent smoking is transitional, experimental, or a longer-term pattern, or whether the function of intermittent smoking differs across groups. Intermittent smoking is more common among younger people, women, those with higher education, and racial/ethnic minorities such as African Americans and Hispanics (Hassmiller et al., 2003; Schane et al., 2010; Trinidad et al., 2009; Wortley et al., 2003). One study reports that Black youths are less likely to transition into daily smoking than White or Hispanic youths (Kandel et al., 2004). Understanding the role of intermittent smoking may provide additional insight into racial/ethnic and gender differences in smoking patterns and trajectories.

We therefore explore whether the relationship between previous (daily and intermittent) smoking and current daily smoking differs by gender and race/ethnicity. Specifically, we consider whether earlier intermittent smoking in young adulthood (Wave III) is associated with daily smoking in early middle adulthood (Wave V), and if so, whether this association varies across gender and racial/ethnic groups. We focus on intermittent smoking in young adulthood because this life stage is when smoking is generally the highest and is just prior to the age crossover pattern described above. Smoking levels for some groups (i.e., Whites and Hispanics) may start decreasing after young adulthood, while increasing for others (i.e., Blacks).

### 1.4 Early life and concurrent influences

In this study, we investigate both early (Wave I) and current (Wave V) characteristics that may shed light on racial/ethnic differences in daily smoking. We identify parental smoking in adolescence, SES in adolescence and at early midlife, and early midlife family structure as potential explanations for gender and racial/ethnic differences in early midlife smoking.

Parental smoking is associated with current smoking in young adults and smoking initiation among adolescents (Gilman et al., 2009; Kandel et al., 2015). Although the effect of parental smoking on adolescent and young adult smoking is found across groups, exposure to parental smoking differs across race/ethnicity and gender (Knight et al., 1996; Muilenburg et al., 2006).

The relationship between smoking and SES has been well established, with lower SES in childhood or adulthood related to a higher chance of smoking (e.g., Hiscock et al., 2012). Lower educational attainment is associated with increased smoking across all racial/ethnic groups, yet educational disparities in smoking have worsened over time, especially among African Americans and Hispanics (Nguyen-Grozavu et al., 2020). However, previous studies have identified persistent racial and ethnic differences in smoking trajectories after adjusting for SES and other variables, suggesting something more than SES is involved (e.g., Lawrence et al., 2014; Pampel, 2008).

Living arrangements may shape life course changes and gender and racial/ethnic variations in smoking. There is heterogeneity in cigarette smoking by marital status and race/ethnicity. For example, cohabitators have the highest smoking prevalence for African Americans, while separated adults have the highest smoking prevalence for Whites (Ramsey et al., 2019). Similarly, having children generally reduces smoking, but this relationship varies by marital status, race/ethnicity, and income (Jun & Acevedo-Garcia, 2007). We therefore consider whether the respondents live with a spouse, children, or parents in early middle adulthood.

### 1.5 Current study

We examine daily and intermittent smoking prevalence across age by gender and race/ethnicity. Based on prior research and our supplementary analyses indicating significant interactions between race/ethnicity and gender in smoking patterns, we separate our multivariate logistic regression models by gender. We seek to answer the following questions:

1) Are there significant gender and racial/ethnic differences in the likelihood of daily smoking in early middle adulthood? Do these differences persist after accounting for early and current sociodemographic characteristics (if any)?
2) Is previous smoking associated with the likelihood of daily smoking in early middle adulthood? If so, does this relationship vary across race/ethnicity and gender?

## 2 Methods

### 2.1 Data

We use nationally representative data from Add Health (Harris et al., 2019), which collected data on various health-related topics throughout five waves: I (1994–1995), II (1996); III (2001–2002); IV (2008–2009), and V (2016–2018). At Wave I, the respondents were adolescents in grades 7 through 12. We focus on daily smoking at the most recent time point, Wave V, a sample that includes 12,300 respondents who were 33–44 years old. Our analytic sample includes 8,506 respondents, limited to non-Hispanic Whites, non-Hispanic Blacks, and Hispanics who have a valid sampling weight, representing the national population of adolescents enrolled in school in 1994–1995. We structure the data at the individual level.

### 2.2 Outcome variable

We measure Wave V daily smoking using respondents’ self-reported smoking frequency during the past 30 days. Those who had smoked 30 of 30 days are categorized as daily smokers (1), whereas those who smoked fewer than 30 days are coded as 0.

### 2.3 Independent variables

Race/ethnicity is represented by mutually exclusive self-identified categories for White, Black, and Hispanic. Those reporting more than one race are assigned to the category they reported as best describing their racial background. Hispanic ethnicity is asked separately from race; thus, respondents reporting Hispanic are coded as Hispanic, and White and Black groups are considered non-Hispanic. Race and ethnicity are taken from Wave I, with Wave III and V data sometimes filling in missing observations. Due to small samples, Asian/Pacific Islander, American Indian/Alaska Native, and “other race” are excluded from analyses.

Gender is self-reported and categorized into males and females. Age at Wave V is presented in years. Respondents’ nativity is coded as U.S.-born = 1 and foreign-born = 0.

We consider the role of prior daily and intermittent smoking.^1^ At Waves I, III, and IV, initial questions asked respondents if they had ever tried cigarette smoking and if so, whether they had ever smoked an entire cigarette. Those who had tried smoking and/or had ever smoked an entire cigarette were asked to report the number of days they smoked during the past month. Following the literature measuring intermittent smoking as non-daily smoking (e.g., Rubinstein et al., 2014; Shiffman et al., 2012; Tindle & Shiffman, 2011; Trinidad et al., 2009), those who reported smoking on 30 days are identified as daily smokers, those who smoked on fewer than 30 days as intermittent smokers, and those who did not smoke at all as nonsmokers. However, Wave III differs from other waves. Those having tried a cigarette are asked if they have ever smoked regularly, and those answering yes are asked to identify the number of days. This skip pattern likely underestimates Wave III intermittent smokers.

Parent education and household income represent adolescent SES. We code parent education into: not completed high school, high school degree, some college, and college degree or higher. We use parent reports from the Wave I parent survey, and if those are unavailable, substitute Wave I adolescent responses on parental education. We average levels for the mother and her spouse/partner or use one measure if only one parent's education is provided. Following Cubbin et al. (2005), parent-reported household income is a percentage of 1994 federal poverty thresholds adjusted for household size. We code this measure as: 0 to 100, 101 to 200, 201 to 300, 301 to 400, and 400% or more. Parent smoking status captures whether the resident mother or father ever smoked, reported by adolescents in Wave I. If the child's responses were missing, the parent response was substituted.

We examine key early midlife factors from Wave V: SES and family structure. Each respondent's highest level of education is recoded into a continuous measure of years. A categorical variable represents personal earnings, with one category for zero earnings and other respondents divided into: $1 - $39,999 (low), $40,000–74,999 (mid), and $75,000 and above (high). A binary variable captures whether the respondent lived with their parents. A categorical variable represents family structure: unmarried without children, married with children, married without children, and unmarried with children.

### 2.4 Analysis

First, we characterize the analytic sample (N = 8,506), including tests for significant differences among Wave V daily smokers, intermittent smokers, and nonsmokers. Smoking prevalence for Waves I, III, IV, V and gender and racial/ethnic subgroup are presented.

Next, we estimate binary logistic regression models split by gender to examine racial/ethnic differences in the odds of daily smoking in early middle adulthood (Model 1). We sequentially add adolescent and early midlife variables to examine whether differences across race/ethnicity persist (Models 2 and 3). To examine the relationship between young adult intermittent smoking and early midlife daily smoking and possible variations across gender and race/ethnicity, we first add the Wave III smoking measure to the base model (Model 4), then interact the smoking measure with race/ethnicity (Model 5). Model 6 includes all independent variables.

We use multiple imputation to address missing values for independent and dependent variables, retain all cases, and reduce bias (Allison, 2002). Analyses are conducted using Stata, with “mi impute chained” imputing missing values (StataCorp, 2011).^2^ All analyses account for complex sampling design, using Add Health variables for longitudinal weights, cluster, and strata to produce nationally representative findings.

## 3 Results

### 3.1 Sample characteristics

Table 1 presents characteristics of the analytic sample. Overall, the sample reflects the national composition of White, Black, and Hispanic adults entering early middle age. Significance tests show that daily smokers, intermittent smokers, and nonsmokers differ. Compared to Wave V daily smokers (reference), intermittent and nonsmokers show more advantages in adolescence and early midlife. For example, a higher proportion of daily smokers (78%) reports either parent ever smoking, followed by intermittent smokers (69%) and nonsmokers (64%). Daily smokers are also underrepresented in the highest family of origin SES categories (e.g., parents having a college degree or 400%+ in household income-to-needs). These adolescent disadvantages continue in early middle adulthood. Compared to both intermittent (53%) and nonsmokers (41%), a larger proportion of daily smokers (64%) are categorized into the low personal earnings group, and their education is the lowest (less than 13 years).

**Table 1:** Weighted means (N = 8,506).

	Overall N = 8,506	Wave V Daily Smoker N = 1,333	Wave V Intermittent Smoker N = 740	Wave V Nonsmoker N = 6,433
Race/Ethnicity				
**Non-Hispanic white**	.71 (.65–.76)	.80 (.75–.85)	.62 (.54–.71)***	.69 (.63–.75)***
Non-Hispanic black	.16 (.12–.21)	.13 (.09–.17)	.23 (.16–.30)**	.17 (.12–.21)*
Hispanic	.13 (.09–.17)	.07 (.04–.09)	.15 (.10–.20)**	.14 (.10–.19)***
Female	.50 (.48–.51)	.46 (.42–.50)	.41 (.36–.46)	.52 (.50–.54)*
Age at Wave V	37.42 (37.18–37.67)	37.48 (37.15–37.81)	37.38 (37.08–37.68)	37.41 (37.17–37.65)
Born in the US	.96 (.95–.97)	.98 (.97–.99)	.96 (.93–.98)+	.96 (.94–.97)**
Wave I variables				
Parent smoking	.67 (.65–.69)	.78 (.75–.81)	.69 (.63–.74)**	.64 (.62–.66)***
Parent education				
**Less than high school**	.21 (.18–.23)	.26 (.22–.30)	.22 (.17–.27)	.19 (.16–.22)**
High school degree	.21 (.20–.24)	.27 (.23–.31)	.20 (.15–.24)**	.20 (.18–.22)***
Some college	.40 (.37–.42)	.40 (.36–.44)	.39 (.33–.45)	.40 (.37–.43)
College degree and higher	.18 (.15–.21)	.07 (.05–.09)	.19 (.14–.24)***	.21 (.17–.25)***
Household income-to-needs				
**Less than 100%**	.17 (.15–.20)	.22 (.18–.26)	.17 (.13–.23)	.16 (.13–.19)**
100–200%	.24 (.22–.25)	.30 (.26–.34)	.25 (.20–.31)	.22 (.19–.24)***
200–300%	.22 (.20–.23)	.20 (.16–.23)	.22 (.17–.26)	.22 (.20–.24)
300–400%	.15 (.14–.17)	.13 (.10–.16)	.15 (.10–.19)	.16 (.14–.18)*
400% +	.22 (.19–.25)	.15 (.12–.18)	.21 (.15–.26)*	.24 (.21–.27)***
Wave V variables				
Years of education	14.33 (14.14–14.52)	12.97 (12.80–13.14)	13.87 (13.56–14.18)***	14.77 (14.58–14.96)***
Personal earnings				
**Low**	.47 (.44–.49)	.64 (.61–.68)	.53 (.47–.59)***	.41 (.38–.43)***
Middle	.31 (.30–.33)	.25 (.22–.27)	.28 (.24–.33)	.33 (.32–.35)***
High	.22 (.20–.24)	.11 (.08–.14)	.18 (.14–.22)**	.26 (.24–.28)***
Live with parents	.08 (.07–.09)	.12 (.09–.14)	.10 (.07–.13)	.07 (.06–.08)**
Family structure				
Married, with children	.49 (.47–.51)	.34 (.30–.37)	.40 (.34–.46)+	.55 (.52–.57)***
Married, no children	.08 (.07–.08)	.06 (.05–.08)	.06 (.03–.09)	.08 (.07–.09)+
Not married, with children	.21 (.19–.23)	.32 (.29–.35)	.25 (.21–.30)*	.18 (.16–.20)***
**Not married, no children**	.22 (.21–.24)	.28 (.25–.31)	.29 (.24–.33)	.19 (.18–.21)***

*Source*: National Longitudinal Study of Adolescent to Adult Health (1994–2018).

Reference groups are bolded.

Significance tests compare intermittent and nonsmoking to daily smoking.

95% Confidence intervals in parentheses.

+ p < .10; * p < .05; ** p < .01; *** p < .001.

### 3.2 Descriptive findings: daily, intermittent, and nonsmoking over the life course

**Fig. 1:**
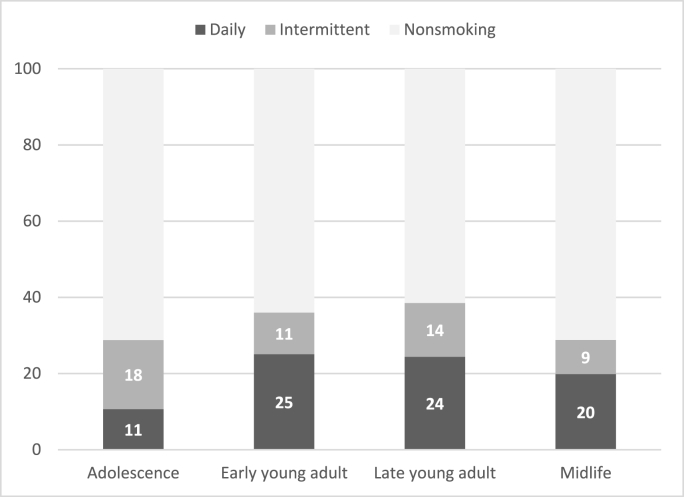
Smoking categories over the life course (N = 8,506) *Source*: National Longitudinal Study of Adolescent to Adult Health (1994–2018). Notes: Adjusted for complex sampling design.

**Fig. 2:**
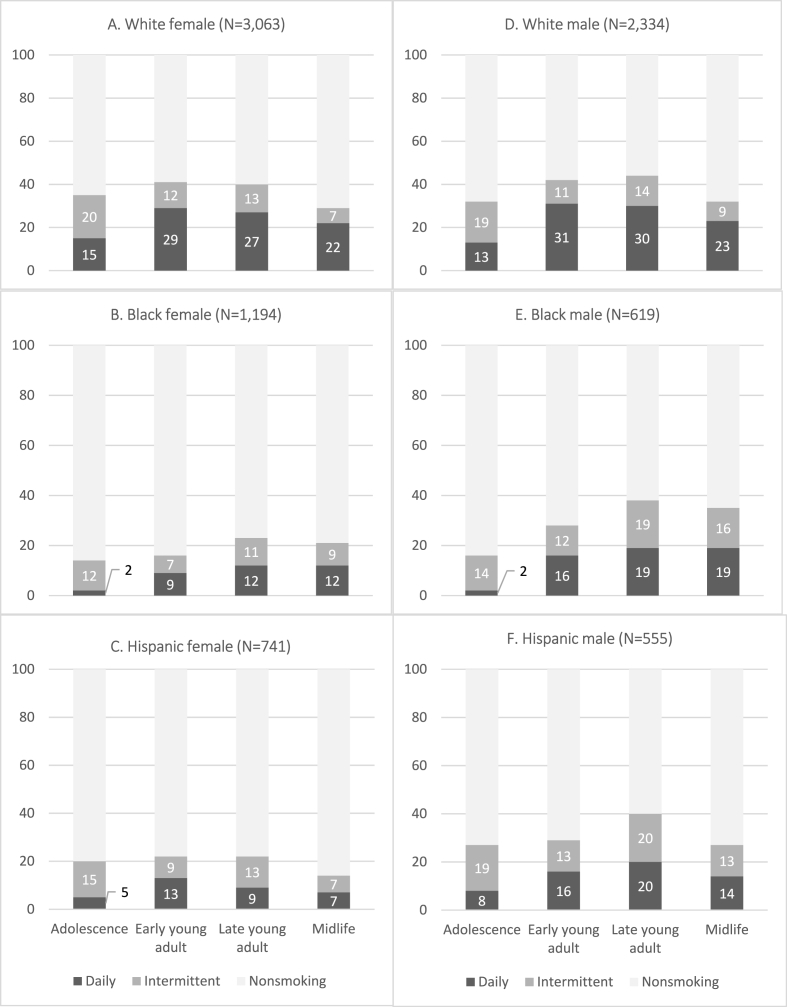
(A–F). Smoking categories over the life course by subgroups *Source*: National Longitudinal Study of Adolescent to Adult Health (1994–2018). Notes: Adjusted for complex sampling design.

Fig. 1, Fig. 2 present daily, intermittent, and nonsmoking prevalence across age for the analytic sample (Fig. 1) and six gender and racial/ethnic subgroups (Fig. 2A–F). As shown in Fig. 1, any smoking (daily or intermittent) is the lowest at Wave I when respondents were in middle or high school. Smoking prevalence increases with age, and any smoking peaks at Wave IV when respondents reach late young adulthood, then decreases substantially at Wave V. In early middle adulthood at Wave V (ages 33–44), smoking prevalence is approximately 29% (daily 20%, intermittent 9%).

Fig. 2 (A–F) shows variation in smoking prevalence over time across gender and racial/ethnic subgroups. White male and female respondents show higher proportions of intermittent than daily smoking at Wave I, but these patterns subsequently reverse, with larger proportions of daily than intermittent smokers. In adolescence, Black respondents' smoking is mostly intermittent, with very low daily smoking (Fig. 2B and E). In early middle adulthood, daily and intermittent smoking are somewhat evenly distributed for Black and Hispanic respondents. At this point, the crossover between White and Black any smoking prevalence is observed for men but not women. Black and Hispanic males show similar trajectories: highest prevalence in late young adulthood with similar proportions of intermittent and daily smokers. Hispanic and White women follow similar trajectories, yet Hispanic women's proportions of daily and intermittent smokers more closely resemble those of Black women.

### 3.3 Racial/ethnic differences in early midlife daily smoking

Table 2 presents logistic regression models predicting women's odds of daily smoking in early middle adulthood. Controlling for age and nativity, non-Hispanic Black and Hispanic women are approximately 50–70% less likely than non-Hispanic White women to smoke daily in early middle adulthood (p < .001; Model 1). Supplemental analysis comparing odds for Black and Hispanic women finds no significant difference.

**Table 2:** Odds ratios and 95% confidence intervals from binary logistic regression models predicting Wave V daily smoking (Female N = 4,998).

	Model 1	Model 2	Model 3	Model 4	Model 5	Model 6
Black	.49 ***	.36 ***	.26 ***	1.16	.66 +	.33 ***
	(.35–.70)	(.25–.53)	(.18–.39)	(.82–1.64)	(.41–1.05)	(.20–.54)
Hispanic	.29 ***	.20 ***	.20 ***	.41 **	.16 **	.10 **
	(.17–.50)	(.11–.37)	(.11–.37)	(.24–.68)	(.04–.61)	(.03–.36)
U.S. born (yes = 1)	1.43	1.48	1.41	1.36	1.19	.99
	(.66–3.12)	(.65–3.38)	(.59–3.35)	(.65–2.83)	(.56–2.54)	(.46–2.15)
Age at Wave V	1.06	1.05	1.06 +	1.07 *	1.07 +	1.08 *
	(.99–1.13)	(.98–1.13)	(.99–1.14)	(1.00–1.15)	(1.00–1.15)	(1.01–1.16)
Wave III intermittent				5.82 ***	4.55 ***	4.19 ***
				(3.88–8.74)	(2.87–7.21)	(2.58–6.79)
Wave III daily				22.01 ***	17.58 ***	13.53 ***
				(15.63–31.00)	(12.30–25.14)	(9.46–19.36)
Black x Wave III intermittent					3.72 **	3.82 **
					(1.68–8.25)	(1.49–9.82)
Black x Wave III daily					2.50 *	3.21 **
					(1.22–5.12)	(1.43–7.21)
Hispanic x Wave III intermittent					1.31	1.20
					(.17–10.18)	(.15–9.80)
Hispanic x Wave III daily					3.93 +	5.58 *
					(.84–18.34)	(1.17–26.55)
**Wave I variables**						
Parent smoking (yes = 1)		1.79 ***	1.64 ***			1.29 +
		(1.42–2.26)	(1.29–2.11)			(.96–1.72)
*Household income-to-needs (<100)*						
100–200		.81	.98			.97
		(.54–1.22)	(.63–1.50)			(.59–1.61)
200–300		.59 *	.81			.81
		(.38–.90)	(.52–1.27)			(.49–1.36)
300–400		.60 *	.96			.98
		(.39–.94)	(.59–1.56)			(.58–1.66)
400 +		.52 **	.82			.81
		(.33–.82)	(.51–1.32)			(.48–1.39)
*Parent education (less than high school)*						
High school degree		.88	1.12			.87
		(.65–1.20)	(.82–1.53)			(.62–1.22)
Some college		.64 **	.97			.76
		(.47–.87)	(.70–1.35)			(.53–1.10)
College degree +		.27 ***	.61 *			.46 **
		(.16–.43)	(.38–.99)			(.27–.77)
**Wave V variables**						
Years of education			.75 ***			.81 ***
			(.71–.80)			(.75–.87)
*Personal earnings (low)*						
Mid			.67 **			.57 **
			(.51–.88)			(.41–.79)
High			.58 *			.65 +
			(.36–.94)			(.42–1.02)
Live with parents			.90			.93
			(.60–1.34)			(.62–1.39)
*Family structure (Not married, no children)*						
Married, with children			.54 **			.61 *
			(.38–.76)			(.41–.92)
Married, no children			.54 *			.57 +
			(.30–.95)			(.32–1.02)
Not married, with children			1.16			1.21
			(.81–1.67)			(.79–1.84)

Source: National Longitudinal Study of Adolescent to Adult Health (1994–2018).

Notes: Adjusted for complex sampling design.

Reference group in parentheses.

+ p < .10; * p < .05; ** p < .01; *** p < .001.

Model 2 reveals that early life characteristics are associated with early midlife smoking, but observed racial/ethnic differences are similar when considering these influences. Early disadvantages, such as parent smoking, lower family SES in adolescence (parent education and household income) predict higher odds of daily smoking, suggesting a prolonged relationship between early life circumstances and later adult smoking. Similarly, after adjusting for early midlife characteristics in Model 3, the racial/ethnic differences persist. These models suggest that early and current sociodemographic characteristics do not account for racial/ethnic differences in early midlife daily smoking for women.^3^

Table 3 presents results from logistic regression models predicting men's likelihood of daily smoking in early middle adulthood. Controlling for age and nativity, there are no significant differences between non-Hispanic White and Black men (Model 1). Hispanic men are approximately 40% less likely to smoke daily than White men (p < .05). Supplemental analyses comparing odds for Black and Hispanic men indicate no significant difference. These results differ from those of women, highlighting the importance of an intersectional perspective.

**Table 3:** Odds ratios and 95% confidence intervals from binary logistic regression models predicting Wave V daily smoking (Male N = 3,508).

	Model 1	Model 2	Model 3	Model 4	Model 5	Model 6
Black	.81	.71 *	.52 ***	1.25	.80	.53 *
	(.66–1.08)	(.53–.96)	(.39–.71)	(.89–1.78)	(.44–1.46)	(.29–.97)
Hispanic	.59 *	.47 **	.44 **	.80	.82	.61
	(.36–.98)	(.27–.80)	(.26–.75)	(.46–1.37)	(.38–1.74)	(.27–1.35)
U.S. born (yes = 1)	1.71	1.92	1.56	1.29	1.29	1.41
	(.76–3.87)	(.85–4.37)	(.67–3.67)	(.55–3.03)	(.54–3.03)	(.48–4.12)
Age at Wave V	1.00	.99	1.00	1.01	1.00	1.01
	(.94–1.07)	(.93–1.05)	(.94–1.06)	(.94–1.09)	(.94–1.08)	(.94–1.08)
Wave III intermittent				3.31 ***	2.69 ***	2.87 ***
				(2.27–4.84)	(1.69–4.27)	(1.81–4.56)
Wave III daily				13.02 ***	11.77 ***	9.34 ***
				(9.78–17.33)	(8.69–15.94)	(6.77–12.90)
Black x Wave III intermittent					3.16 *	2.16
					(1.17–8.55)	(.77–6.03)
Black x Wave III daily					1.81	1.88
					(.84–3.91)	(.83–4.26)
Hispanic x Wave III intermittent					.78	.75
					(.21–2.85)	(.20–2.81)
Hispanic x Wave III daily					1.02	1.08
					(.40–2.59)	(.36–3.23)
**Wave I variables**						
Parent smoking (yes = 1)		1.55 **	1.36 *			1.24
		(1.16–2.06)	(1.02–1.82)			(.89–1.74)
*Household income-to-needs (<100)*						
100–200		.99	1.19			1.03
		(.69–1.41)	(.80–1.77)			(.66–1.61)
200–300		.61 *	.82			.72
		(.41–.91)	(.53–1.27)			(.45–1.16)
300–400		.53 **	.79			.68
		(.34–.83)	(.50–1.26)			(.41–1.14)
400 +		.51 **	.82			.76
		(.33–.78)	(.51–1.30)			(.45–1.28)
*Parent education (less than high school)*						
High school degree		.97	1.24			1.12
		(.66–1.42)	(.83–1.85)			(.72–1.75)
Some college		.82	1.29			1.28
		(.59–1.14)	(.94–1.76)			(.89–1.83)
College degree +		.31 ***	.72			.65
		(.19–.50)	(.44–1.19)			(.36–1.16)
**Wave V variables**						
Years of education			.73 ***			.77 ***
			(.68–.79)			(.70–.85)
*Personal earning (low)*						
Mid			.73 *			.73 +
			(.55–.97)			(.53–1.01)
High			.56 **			.66 *
			(.39–.81)			(.44–.99)
Live with parents			.96			1.09
			(.59–1.57)			(.63–1.88)
*Family structure(Not married, no children)*						
Married, with children			.45 ***			.44 ***
			(.32–.64)			(.30–.65)
Married, no children			.72			.75
			(.45–1.17)			(.46–1.21)
Not married, with children			.98			.94
			(.67–1.42)			(.62–1.41)

Source: National Longitudinal Study of Adolescent to Adult Health (1994–2018).

Notes: Adjusted for complex sampling design.

Reference group in parentheses.

+ p < .10; * p < .05; ** p < .01; *** p < .001.

Model 2 adjusts for early life characteristics. The difference in daily smoking between Hispanic and White men persists with a larger magnitude, and there is a significant difference between Black and White men at p < .05. Interestingly, the race/ethnicity gaps remain after adjusting for early midlife characteristics in Model 3.

We now turn to prior smoking. For women, Model 4 in Table 2 shows that both intermittent and daily smoking in early young adulthood (Wave III) are related to much higher odds of daily smoking in early middle adulthood. Notably, there is no significant difference between White and Black women in the likelihood of early midlife daily smoking when previous smoking is adjusted in Model 4. Model 5 interacts race/ethnicity with Wave III smoking categories to identify whether there is heterogeneity in the association with subsequent daily smoking. We find statistically significant and fairly strong positive interaction effects indicating that Wave III intermittent and daily smoking predict an increased likelihood of Wave V daily smoking disproportionately more for Black women than White women. These results further persist in Model 6, which includes all covariates.

For men, previous intermittent or daily smoking is also associated with much higher odds of daily smoking in early middle adulthood (Model 4, Table 3). Further, the racial/ethnic smoking disparities for men look different when considering prior smoking: there are no longer any significant differences between White and Hispanic men (Model 4, Table 3). Consistent with findings for women, we find a significant interaction effect for Black x Wave III intermittent smoking (Model 5, Table 3). That is, while young adult intermittent smoking is associated with a higher likelihood of daily smoking in early midlife, this relationship is stronger for Black individuals. As compared to Whites, Black men have a disproportionately greater chance of transitioning from intermittent to daily smoking from early young to early middle adulthood. However, this difference does not persist for men once we consider all covariates in Model 6.

## 4 Discussion

In this study, we identified racial/ethnic and gender differences in early midlife smoking using a recent nationally representative US sample. We find evidence that the odds of early midlife daily smoking differ across race/ethnicity and gender subgroups. These differences persist after considering early and current sociodemographic characteristics. We also find that while prior daily or intermittent smoking is a strong predictor for daily smoking in early midlife, the effect of intermittent smoking varies across race/ethnicity and gender. Overall, our study suggests that smoking research and policies should consider a greater diversity of life course patterns in smoking to understand how smoking habits develop over time across social groups and social-ecological contexts. Importantly, the intersection of race/ethnicity, gender, and age can shed light on the social determinants of smoking disparities. We further expound on four key conclusions.

First, our study documents and helps explain differences in smoking uptake over the life course. White women and men tend to start smoking in adolescence and early young adulthood, with rates generally declining in late young and early middle adulthood. In contrast, Black women and men tend to take up smoking in late young adulthood, with smaller declines in early middle adulthood. Hispanic men and women appear to display earlier timing, similar to that of White women and men. Notably, smoking declines for everyone as they approach early middle adulthood but less so for Black women and men. These differences in the timing of smoking demonstrate the importance of examining disaggregated smoking trajectories. The pooled results reflect the majority groups of White men and women, obscuring heterogeneity. Prevention efforts drawing on studies that conclude adolescence is a key life course stage for tobacco uptake may be prioritizing the smoking patterns of White youths. Future interventions could consider young adulthood as a key life course stage for smoking uptake among African Americans. In fact, recent studies reveal that smoking onset is becoming more concentrated in young adults, a population segment once considered beyond the critical risk period for smoking onset (Barrington-Trimis et al., 2020; Cantrell et al., 2018; Thompson et al., 2018). Considering this recent shift in smoking onset and heterogeneity across race/ethnicity, expanding the emphasis on prevention to include young adults is imperative.

Second, we find racial/ethnic and gender differences in early midlife daily smoking. In this life stage, there is not a significant difference between White and Black men, yet White men are more likely to smoke daily than Hispanic men. On the other hand, White women are more likely to smoke daily than both Black and Hispanic women. There is no significant difference between White and Black women or between White and Hispanic men when prior smoking is adjusted. These results suggest that early young adult smoking plays an important role in understanding the development of smoking disparities across race/ethnicity. In addition, although White women are at the greatest risk for daily smoking in early middle adulthood, our descriptive findings (Fig. 2A–C) suggest that the gap between White and Black women may disappear with age if the smoking patterns continue. If smoking prevalence continues decreasing for White women while stagnating for Black women, we may see the crossover point when respondents are in late middle adulthood. Just as White and Black men demonstrate similar odds of daily smoking in early middle adulthood, we may see the same pattern in women a few years later. Given increasing and stable smoking prevalence over time for African Americans, future interventions should also consider smoking cessation efforts for middle-aged smokers, especially Black women.

Third, we find that patterns of intermittent and daily smoking differ across subgroups. Intermittent smoking is particularly prevalent among Black and Hispanic men in late young and early middle adulthood, and Black and Hispanic women also display greater or similar proportions of intermittent compared to daily smoking at these ages compared to their White peers. This finding is in line with current literature reporting that light or intermittent smoking is common among minority populations, including Black and Hispanic smokers (Schane et al., 2010). Additionally, both intermittent and daily smoking in young adulthood are associated with daily smoking in early middle adulthood, and the association between young adult intermittent smoking and early midlife daily smoking is particularly strong for Black women, suggesting that the social meaning of intermittent smoking may differ across race/ethnicity and gender.

Fourth, we argue that intermittent smoking should be an important component of future tobacco research and policy. Intermittent smoking may reflect experimentation in some younger individuals but also constitute a longer-term pathway to daily smoking. Thus, intervention efforts could consider preventing not just uptake but also progression from intermittent to daily smoking (Villanti et al., 2019). Although daily smoking has the greatest health risks, any smoking is associated with increased health risks (Gawlik, Melnyk, & Tan, 2018), and light and intermittent smoking may carry similar cardiovascular risk to daily smoking (Clawson et al., 2021; Schane et al., 2010). Smoking patterns (e.g., higher early intermittent smoking and progression into later daily smoking) and associated health risks highlight the need for refocusing intervention efforts on light or intermittent smokers. Research on smoking disparities has largely focused on daily smoking, but intermittent smoking is increasingly common and reflects changing social norms and smoking behaviors (Shiffman, 2009). Future research should consider intermittent alongside daily smoking to better capture tobacco exposure and its social causes.

This study has several limitations. We conducted associational analyses and are thus limited in making causal inferences. Similarly, we did not conduct formal mediation analysis to identify mechanisms for racial/ethnic and gender differences. Future research could examine how smoking probability disparities by race/ethnicity and gender shift across models for mediation analyses. Smoking categories could further be disaggregated, possibly by days of smoking in the past month and number of cigarettes smoked per day for daily smokers, offering more elaborate insight into life course smoking patterns. It would be interesting to examine smoking transitions in greater detail; for instance, racial/ethnic and gender differences in the transitions between daily to intermittent or intermittent to nonsmoking across each stage over the life course. Unfortunately, sample sizes did not allow us to document trajectories for other racial/ethnic groups. We hope that future research will examine patterns for Asian/Pacific Islanders, Native Americans, and multiracial individuals.

An intersectional life course perspective suggests interventions should consider greater heterogeneity in smoking patterns. Smoking differs across the intersections of race/ethnicity, gender, and age. Thus, understanding life course patterns can help to shed light on how and why people from diverse backgrounds and complex experiences start, stop, increase, or decrease smoking.

## Ethical statement

We have reviewed the Ethics in Publishing and Ethical Guidelines for Journal Publication documents and confirm that we have abided by all ethical guidelines in the production of this manuscript. We have no competing interests or financial interests to disclose.

## Author contributions

Juhee Woo: Conceptualization, Methodology, Analysis, Visualization, Writing - Original Draft, Writing - Reviewing and Editing.

Elizabeth Lawrence: Conceptualization, Methodology, Writing - Original Draft, Writing - Reviewing and Editing.

Stefanie Mollborn: Conceptualization, Methodology, Writing - Reviewing and Editing.

## Declaration of competing interest

The authors declare no conflicts of interest.

## Footnotes

1 We exclude Wave II smoking measures from our analysis because Wave II data were collected only one year after Wave I and were restricted to a subsample of respondents.

2 Of the variables used for the 20 imputed datasets, Wave I household income (22%) had the highest percentage of missing information among the eligible cases (N = 8,506). All other variables had less than 4% of missing information. Wave V daily smoking and smoking measures at other waves had less than 1% missing.

3 In supplemental analyses, we added measures of the other substances (e.g., frequent drinking, marijuana and other tobacco products) at Wave I and Wave V. Cigarette smoking is closely related with other substance use as part of people's health lifestyles (Lawrence et al., 2017), which are patterned by gender and race/ethnicity. However, results from these supplemental models are similar to what we present in Table 2, Table 3 and do not alter our conclusions, so we present the more parsimonious models here.
